# Transcriptional profiles define drug refractory disease in myeloma

**DOI:** 10.1002/jha2.455

**Published:** 2022-05-09

**Authors:** Yuan Xiao Zhu, Laura A. Bruins, Xianfeng Chen, Chang‐Xin Shi, Cecilia Bonolo De Campos, Nathalie Meurice, Xuewei Wang, Greg J. Ahmann, Colleen A. Ramsower, Esteban Braggio, Lisa M. Rimsza, A. Keith Stewart

**Affiliations:** ^1^ Division of Hematology‐Oncology Mayo Clinic Phoenix Arizona USA; ^2^ Division of Biomedical Statistics and Informatics, Department of Health Science Research Mayo Clinic Rochester Minnesota USA; ^3^ Department of Laboratory Medicine and Pathology Mayo Clinic Phoenix Arizona USA; ^4^ Division of Medical Oncology and Hematology Princess Margaret Cancer Centre Toronto Ontario Canada

**Keywords:** drug resistance, gene expression, myeloma

## Abstract

Identifying biomarkers associated with disease progression and drug resistance are important for personalized care. We investigated the expression of 121 curated genes, related to immunomodulatory drugs (IMiDs) and proteasome inhibitors (PIs) responsiveness. We analyzed 28 human multiple myeloma (MM) cell lines with known drug sensitivities and 130 primary MM patient samples collected at different disease stages, including newly diagnosed (ND), on therapy (OT), and relapsed and refractory (RR, collected within 12 months before the patients’ death) timepoints. Our findings led to the identification of a subset of genes linked to clinical drug resistance, poor survival, and disease progression following combination treatment containing IMIDs and/or PIs. Finally, we built a seven‐gene model (MM‐IMiD and PI sensitivity‐7 genes [IP‐7]) using digital gene expression profiling data that significantly separates ND patients from IMiD‐ and PI‐refractory RR patients. Using this model, we retrospectively analyzed RNA sequcencing (RNAseq) data from the Mulltiple Myeloma Research Foundation (MMRF) CoMMpass (*n* = 578) and Mayo Clinic MM patient registry (*n* = 487) to divide patients into probabilities of responder and nonresponder, which subsequently correlated with overall survival, disease stage, and number of prior treatments. Our findings suggest that this model may be useful in predicting acquired resistance to treatments containing IMiDs and/or PIs.

## 1 INTRODUCTION

The introduction of immunomodulatory drugs (IMiDs) and proteasome inhibitors (PIs) changed the therapeutic paradigm for treatment of multiple myeloma (MM) due to their specific antimyeloma mechanisms. While the majority of patients receiving combination chemotherapy including IMiD and/or PI initially respond, most of them eventually develop resistance. Understanding the underlying mechanisms of nonresponsiveness and identifying biomarkers associated with drug resistance and disease progression have become critical for personalized medicine and development of novel therapeutic strategies.

IMiDs mediate anti‐MM effects by binding to the E3 ubiquitin ligase cereblon (CRBN) [1, 2, 3], which subsequently increases degradation of the transcription factors Ikaros (IKZF1) and Aiolos (IKZF3), culminating in downregulation of *IRF4* and *MYC* expression leading to inhibition of MM cell growth [4, 5]. IMiD resistance in MM has been linked to deletion, functional mutation, or dysregulation of CRBN and the proteins directly and indirectly associated with CRBN or IMiD‐mediated signaling [2, 6, 7, 8, 9, 10, 11, 12, 13, 14]. Resistance to PIs in MM has also been extensively studied [15, 16, 17, 18, 19, 20, 21] and attributed to mutation and dysregulation of proteasome subunits [15] .

In clinical practice, IMiDs and PIs are usually given in combination with each other as well other classes of drugs (such as dexamethasone) as standard of care. Developing a method to monitor both IMiD and PI drug sensitivity and detect disease progression during treatment is important for precision medicine. In the present study, we sought to identify transcriptional changes associated with treatment nonresponse by studying the candidate genes previously associated with IMID and/or PI sensitivity to determine whether measuring expression levels of such genes could serve as a biomarker for disease progression.

NanoString nCounter technology, a direct multiplexed measurement of gene expression based on digital color‐barcoding technology [22], is a flexible, reproducible, and robust method when used for molecular subtyping of diffuse large B‐cell lymphoma [23, 24, 25]. In this study, we employed this technology to investigate the transcriptional expression of 121 genes potentially associated with IMiD or PI response, in both human myeloma cell lines (HMCLs) and primary MM patient samples collected at different times of the disease.

## 2 MATERIAL AND METHODS

### 2.1 Study design

**FIGURE 1:**
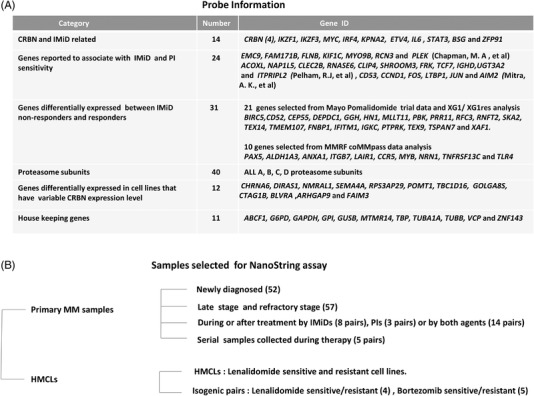
Collated gene list and primary multiple myeloma (MM) samples or human multiple myeloma (MM) cell lines (HMCLs) for NanoString profiling. (A) Genes comprising the CodeSet were selected based on previous studies. (B) Patient materials were selected and grouped based on the stage of disease activity when samples were collected. Numbers in brackets indicate the number of probes for each gene or number of patients in each group

We investigated the transcriptional expression of 121 genes selected from published literature [2, 4, 7, 14, 26–33] and an in‐house database linked to IMiD and PI response and resistance (Figure 1A). We screened 28 HMCLs with known drug sensitivities to IMiDs and PIs and 156 primary MM patient samples, including 41 patients with paired samples, collected at various stages of disease evolution, including newly diagnosed and untreated (ND), collected on therapy (OT), and late relapsed and refractory (RR, bone marrow samples from treated patients taken within the 12 months preceding their death) (Figure 1B, Supporting Information Data 1). Differential gene expression between sample groups with expected distinct drug response profiles and disease stages (sensitive vs. resistant HMCLs and ND vs. during treatment or vs. RR patient samples) were analyzed.

### 2.2 MM cell lines and human MM cells

All HMCLs used in this study were provided by the Bergsagel laboratory and fingerprinted to confirm their identity [34]. The cells were cultured in RPMI1640 medium with 5% fetal calf serum. Isogenic IMID and PI‐sensitive and ‐resistant cell lines were previously generated in our laboratory [7, 33]. The generation of OCIMY5/Vec and OCIMY5/CRBN by coculture with drug was also previously described [28].

Primary human MM cells were recovered from bone marrow aspirates collected from all Mayo Clinic sites. Informed consent was given in writing for collection and research use under Institutional Review Board approval (IRBs 919‐04, 15–009436, 18–003198, 2207‐02) in accordance with the Declaration of Helsinki. After collection, CD138+ cells were isolated by immunomagnetic bead selection (RoboSep; Stemcell Technologies).

### 2.3 Sample preparation and RNA extraction

Total RNA from HMCLs and CD138‐selected plasma cells from MM patient bone marrows were isolated using RNeasy Mini kit and the AllPrep DNA/RNA Kit (Qiagen), respectively. After spectrophotometric quantification (NanoDrop2000, Thermo‐Fisher Scientific), RNA was stored at −80°C until use.

### 2.4 Collated gene list

A unique list including 121 gene candidates was selected for NanoString profiling (Figure 1A). We first selected 26 probes targeting *CRBN*, genes with altered transcription in cell lines with low versus normal *CRBN* expression, genes encoding proteins associated with CRBN activity [26], or additional genes linked to IMiDs activity and sensitivity (such as *IKZF1*, *IKZF3*, *IRF4*, and *MYC*). Since *CRBN* isoforms, including the isoform lacking exon 10, have been associated with IMiD sensitivity [35], four probes targeting different exon junctions of *CRBN* were included, with one (*CRBN* 3) designed to span the exon 10/11 junction. Twenty‐one genes were selected by analyzing baseline gene expression levels associated with drug response in a cohort of 44 refractory MM patients before initiation of pomalidomide and dexamethasone therapy on a phase 2 clinical trial [28, 36, 37] (Supporting Information Data 2) and from the isogenic lenalidomide‐sensitive/resistant HMCL XG1 pair (XG1/XG1LenRes) with normal CRBN levels [7] (Geo123506). Twenty of these gene targets were identified between the responders and nonresponders in both data sets.

An additional 10 genes included in the exploratory panel were selected based on data from 59 MM patients that exhibited differential responses to a first line of treatment containing IMiDs in the CoMMpass data (Supporting Information Data 3, generated as part of the Multiple Myeloma Research Foundation Personalized Medicine Initiatives). These 10 genes were differentially expressed in samples that showed complete response, partial response or stable disease; five of which were also identified in the XG1/XG1LenRes analysis. Additional previously reported 24 genes noted as predictive markers of response to IMiDs and/or PIs in MM were included [30, 31, 32]. Since a recent study in our laboratory identified an upregulation of proteasome subunit genes in PI‐resistant cell lines when compared with their isogenic‐sensitive cell lines [33], 40 probes for genes encoding the multiple proteasome subunits were included.

### 2.5 NanoString CodeSet design and expression quantification

The 121informative genes potentially associated with IMiD and PI response, along with 11 housekeeping genes (Supporting Information Data 4), were combined to generate the exploratory CodeSet for this study. The experiments were performed with nCounter Elements XT reagents in accordance with the manufacture's recommendations. An input of 100 ng total RNA was used for all lines and samples. The collected data were first evaluated for quality control, followed by technical normalization using synthetic controls and biological normalization via housekeeping genes. Data were initially analyzed using nSolver 4.0 software and an advanced analysis software plugin (version 2.0, R‐based statistical tool) to detect and visualize differentially expressed genes.

### 2.6 Ranking predictive probes and development of models for predicting drug sensitivity and disease progression

Differentially expressed genes between ND (*n* = 51) and RR samples (*n* = 57) were selected using edgeR [38]. The expression correlations between the differentially expressed genes were shown by Pvclust [39]. Each gene was then individually analyzed using a generalized linear model [40] to filter the top q‐associated probes with different response outcomes. To build a multivariate ordinal model for prediction, with the 121 gene probes and annotated ND or RR for each patient sample, a linear logistic regression model was built by using R package bhGLM [40], followed by step Akaike information criterion (AIC) [41] for optimization. The resulting prediction model was based on gene expression from seven genes using following calculation:

1. *Z* = −32.1129 + 0.3773DIRAS1 + 2.2558CRBN_2 + 0.8810CD53 + 1.6225PSMD14 – 0.6304CEP55 + 0.9362SK2 ‐ 1.7078PSMA7
2. Based on the value *Z*, the probability of “responder” is calculated by sigmoid function
$$\mathrm{Probability}\;\mathrm{of}\;\mathrm{responder}=\frac{1}{1+e^{-z}}$$

The predicted probability from the model ranges from 0 to 1, where a higher value meant a higher chance of being a responder.

### 2.7 Assessment of the performance of established model

We first evaluated the performance of seven‐gene model (named MM‐IP‐7, stands for multiple myeloma‐IMiD and PI sensitivity ‐7 genes) by five‐fold cross‐validation as described (https://rdrr.io/cran/cvAUC/man/ci.cvAUC.html). Using this model, we retrospectively analyzed existing RNA sequencing (RNAseq) data from the primary samples of the Multiple Myeloma Research Foundation (MMRF) coMMpass (*n* = 578, ND patients) and Mayo Clinic MM registry (*n* = 487, collected from patients at different disease stages) datasets to correlate the MM‐IP‐7 results with other clinical data such as survival, disease stage, and number of treatment protocols. Briefly, we submitted RNAseq data to MM‐IP‐7 to calculate probabilities (by ranking scores) and then compared estimated results with other clinical data in each dataset. Since the RNAseq data are highly correlated but have different scales when compared to NanoString, the probability of estimate from this analysis is based on rank order rather than actual cut point criteria.

## 3 RESULTS

### 3.1 Validation and quality control of NanoString expression profiling in MM

**FIGURE 2:**
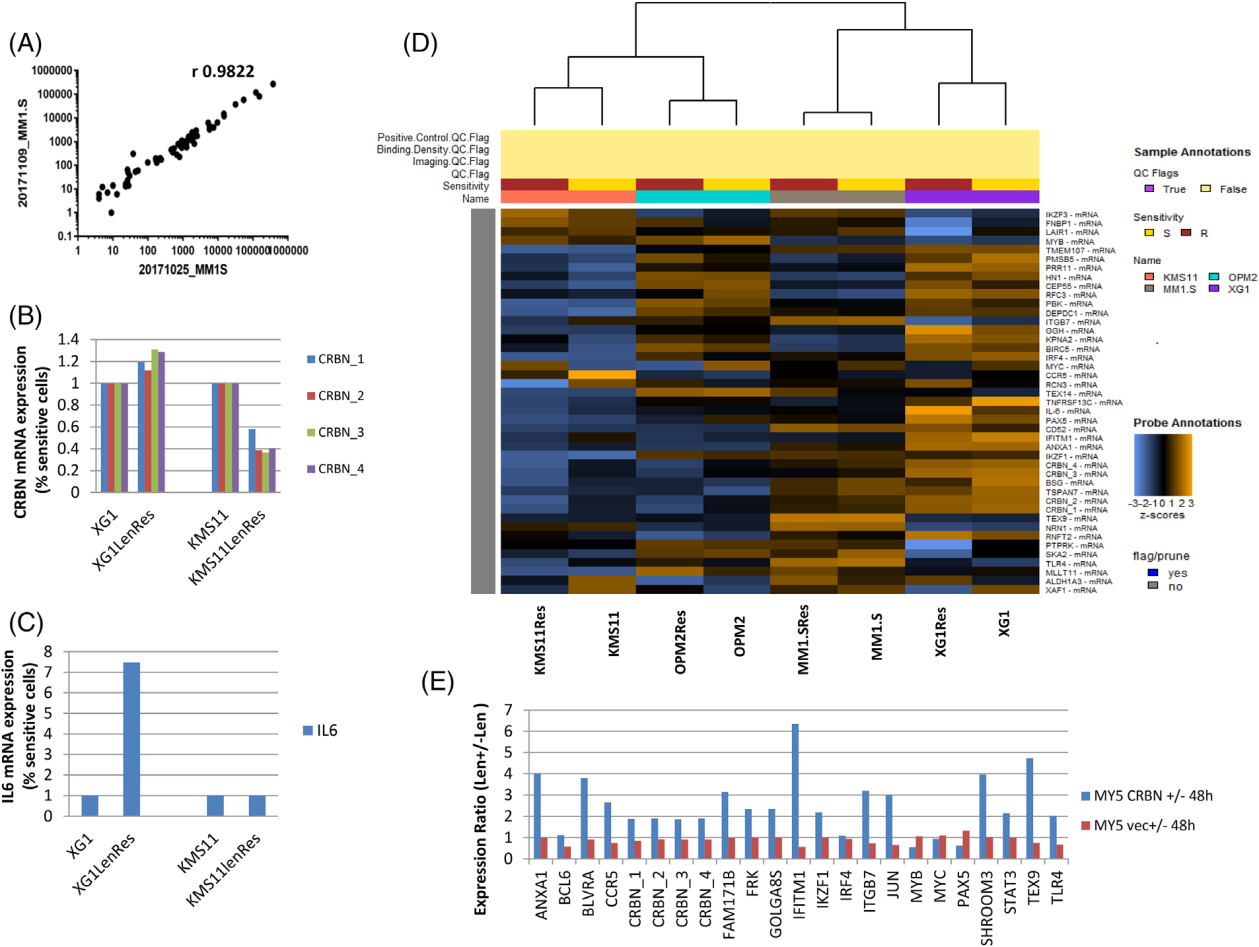
Demonstrating NanoString technology as a sensitive, reliable and reproducible method to quantitate gene expression changes in myeloma cells. (A) Correlation of two biological repeats generated from the NanoString profiling of multiple myeloma1 (MM1).S cell lines. (B and C) NanoString profiling detected the downregulation of *CRBN* mRNA and upregulation of *IL6* mRNA in two different lenalidomide isogenic‐resistant cell lines, consistent with the previous RNA sequencing (RNAseq) data. (D) Heatmap view of the normalized data from four pairs of isogenic introduction of immunomodulatory drugs (IMiDs)‐sensitive/resistant cell lines. (E) Detection of lenalidomide‐mediated transcriptional response in lenalidomide‐sensitive cell line, OCIMY5/Cereblon (CRBN)

We first confirmed performance by the nCounter instrument by profiling of MM cells using testing a CodeSet of 43 of the 121 selected genes (Supporting Information Data 5). We demonstrated that nCounter technology was able to generate reproducible results from two biological repeats (MM1.S, Figure 2A). As expected, the NanoString platform also detected *CRBN* downregulation and *IL6* upregulation in two established lenalidomide‐resistant HMCLs, when compared with their isogenic‐sensitive cell lines (Figure 2B,C), consistent with previous observations [7]. A gene expression heatmap of normalized data from four pairs of lenalidomide isogenic HMCLs showed that each isogenic cell line pair clustered together as expected. Further analysis of the expression data using the nSolver 4.0 software (NanoString, Seattle, WA) identified downregulation of *CRBN* as a significant change in three resistant cell lines (Figure 2D, Figure S1), consistent with previously published data [7].

Then, using the complete CodeSet, we measured the relative expression levels of the 121 selected genes in untreated and lenolidomide‐treated OCIMY5/vec, which expresses a very small amount of CRBN and is resistant to IMiDs and OCIMY5/*CRBN*, which has forced CRBN expression and is sensitive to IMiDs. We were able to accurately detect known lenalidomide‐regulated gene expression changes (Figure 2E) in OCIMY5/*CRBN*. Therefore, NanoString technology was confirmed as a sensitive and reproducible method to quantitate gene expression changes in MM cells.

### 3.2 Identification of differentially expressed genes in MM patients with samples collected at different time

**FIGURE 3:**
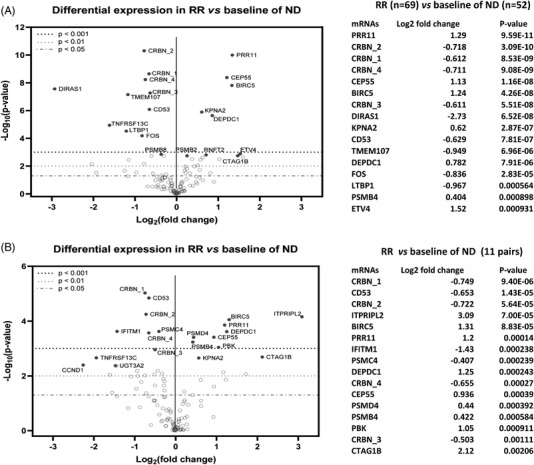
Detection of the differentially expressed genes between newly diagnosed and late stage, relapsed/refractory samples. Volcano plot displaying each gene's ‐log_10_ (*p*‐value) and log_2_ fold change with the selected covariate. Highly statistically significant genes fall at the top of the plot above the horizontal lines, and highly differentially expressed genes fall to either side. Horizontal lines indicate various *p*‐value thresholds. The 20 most statistically significant genes are labeled in the plot. Top 16 differentially expressed genes are shown in the table beside each plot. (A) Fifty late/ relapsed and refractory (RR) samples (bone marrow samples taken from treated patients within the 12 months preceding their death) were compared with 52 newly diagnosed samples. (B) Eleven paired late/RR samples and newly diagnosed (ND) samples were compared

We next measured the differential expression of the 121 genes in all primary MM samples and HMLCs, grouped by known or likely drug sensitivity and resistance profiles. Forty‐three genes were identified that had significantly different expression (*p* ≤ 0.05) between 52 ND and 57 RR samples (Supporting Information Data 6). In addition to the expected *CRBN*, we identified six genes (*TMEM107, DIRAS1, CD53, TNFRSF13C, LTBP1, and FOS*) as most significantly downregulated in RR samples; meanwhile another seven genes (*PRR11*, *CEP55, BIRC5, KPNA2*, *DEPDC1, PSMB4, and ETV4*) were shown as most significantly upregulated (Figure 3A, Supporting Information Data 6). We noticed that all probes against the different *CRBN* isoforms detected CRBN downregulation in RR samples, but no upregulation of the isoform with exon 10 deletion was identified.

**FIGURE 4:**
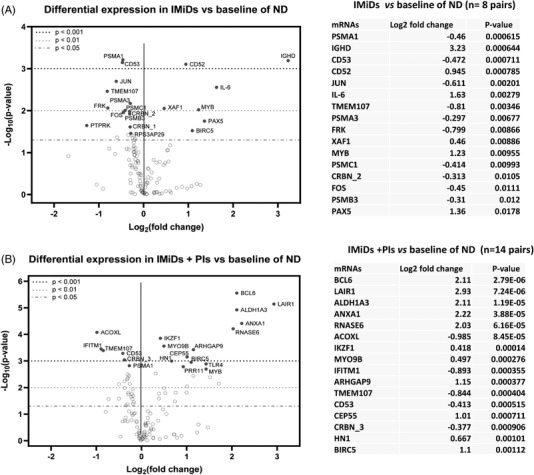
Detection of differentially expressed genes between newly diagnosed multiple myeloma (MM) and samples harvested during active treatment volcano plot displaying each gene's ‐log_10_ (*p*‐value) and log_2_ fold change with the selected covariate. Highly statistically significant genes fall at the top of the plot above the horizontal lines, and highly differentially expressed genes fall to either side. Horizontal lines indicate various *p*‐value thresholds. The 20 most statistically significant genes are labeled in the plot. Top 16 differentially expressed genes are shown in the table. (A) Eight paired samples harvested at the time of diagnosis and during or after treatment with introduction of immunomodulatory drugs (IMiDs)‐based therapy (no proteasome inhibitors [PIs] were used) were compared. (B) Fourteen paired samples harvested at the time of diagnosis and during or after treatment with IMiDs and PIs were compared

We then analyzed 22 paired samples from 11 patients, which were collected at both ND and RR stages, identifying 45 genes as differentially expressed between ND and RR stages (*p* ≤ 0.05, Supporting Information Data 7). In addition to confirming most changes above, the RR samples also showed downregulation of *IFITM1* and *PSMC4* and upregulation of *ITPRIPL2, PBK, PSMD4*, and *CTAG1B* as their most differentially expressed genes (Figure 3B, Supporting Information Data 7). These changes were not detected, or detected at a lower significance, when comparing paired samples at ND with a secondary sample collected during treatment but before disease progression; this dataset included samples from patients treated with IMiDs (eight pairs, Figure 4A and Supporting Information Data 8), IMiDs + PI (14 pairs, Figure 4B and Supporting Information Data 9), and solely PIs (three pairs, Figure S2). When comparing ND samples with paired “on active treatment” samples, downregulation of *CRBN*, *CD53, IFITM1* and upregulation of *PRR11*, *CEP55*, and *BIRC5* was demonstrated (Figure 4B). A similar trend of upregulation of *PRR11*, *ETV4*, and *BIRC5* was also identified in later relapsed samples compared with early samples collected during treatment from five patients (Figure S3).

We next explored HMCLs with known response to IMiDs and PIs [42]. By comparing gene expression of six IMiD‐sensitive and nine IMiD‐resistant HMCLs, 22 genes were identified as differentially expressed (Supporting Information Data 10, Figure S4A). Six changes in IMiD‐resistant HMCLs were consistent with those identified in RR samples from MM patients, including upregulation of *PRR11*, *HN1*, *RFC3*, *PSMB2*, *PSMD14*, and downregulation of *SKA2*. When five paired PI‐sensitive and ‐resistant isogenic cells lines were compared, we identified changes in the expression of seven proteasome subunit genes, including upregulation of *PSMB5* in resistant cell lines (Figure S4B), consistent with our previous observations [33].

### 3.3 Identifying predictive probes and establishing a predictive model

**FIGURE 5:**
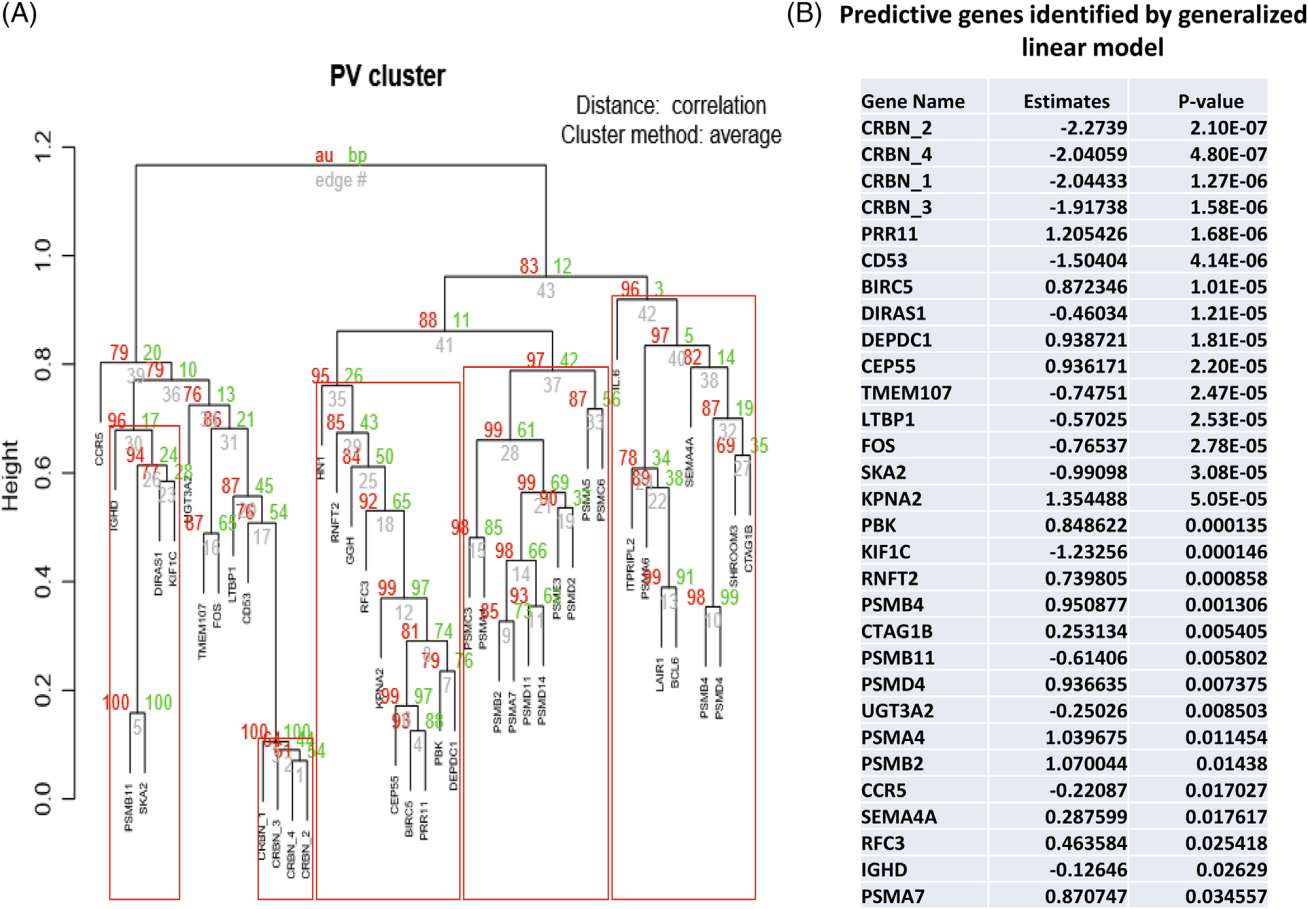
Hierarchical clustering of 45 differentially expressed genes between newly diagnosed (ND) and late/ relapsed and refractory (RR) samples and identification of predictive probes. (A) The expression pattern of 45 differentially expressed genes between the ND and late/RR samples (*p* ≤ 0.01) were analyzed by pvclust. Values at branches are approximately unbiased (AU) *p*‐values (red) and bootstrap probability (BP) values (green). Clusters with AU ≥ 90 are indicated by the rectangles. (B) Predictive genes were identified by analysis of 45 differentially expressed genes between the ND and late/RR samples (*p* ≤ 0.01) using single gene GLM model regression with coefficient *p*‐value ≤ 0.05

**FIGURE 6:**
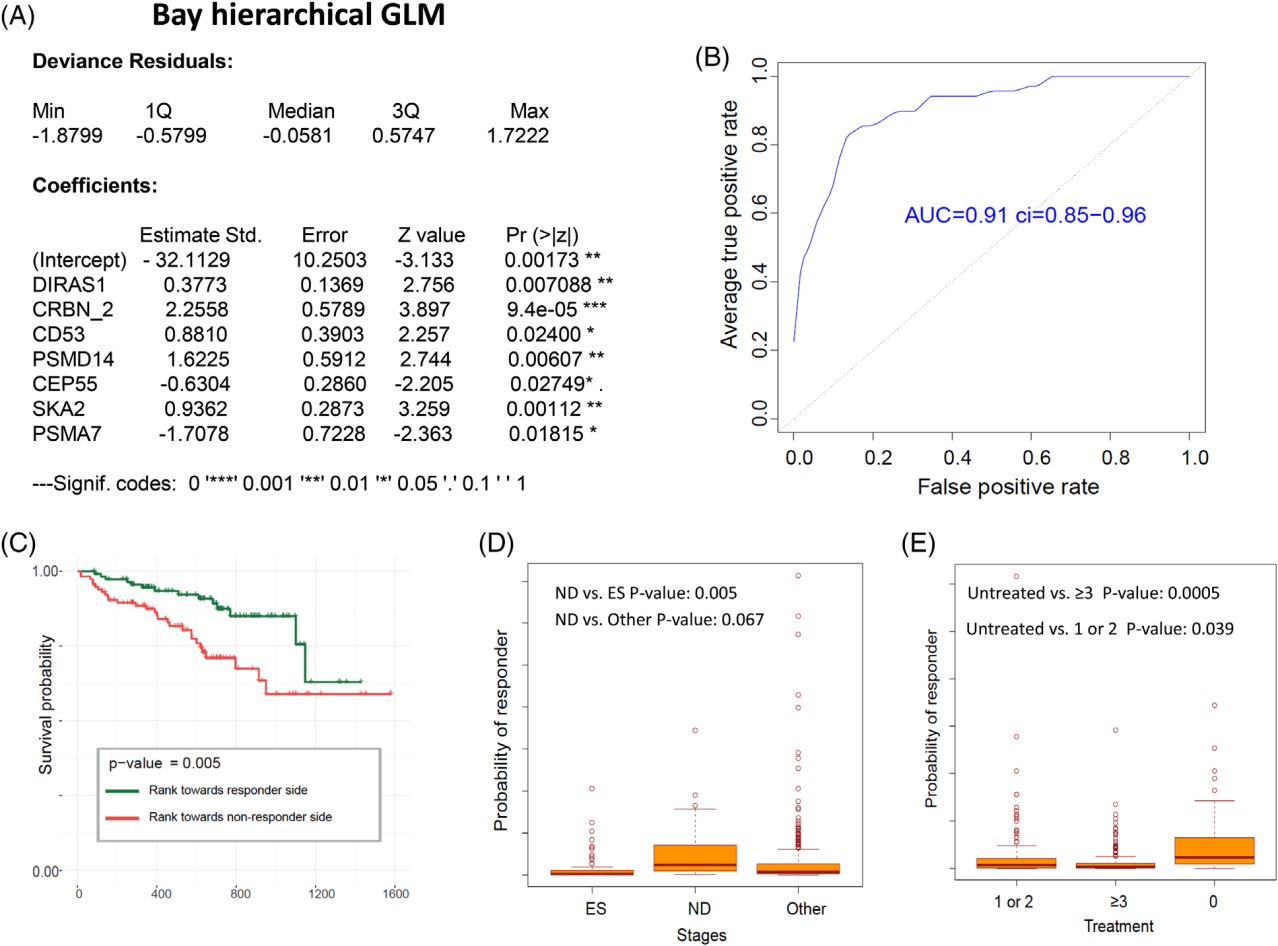
Establishing the predictive model based on the differentiated expressed genes between newly diagnosed (ND) and Late/relapsed and refractory (RR) samples. (A) A seven‐gene predictive model (multiple myeloma [MM]‐IMiD and PI sensitivity‐7 genes [IP]‐7) was built based on a linear logistic regression with R package BhGLM. (B) Area under curve (AUC) plot with 95% confidence interval resulted from five‐fold cross‐validation of established model. (C) The established model was employed on RNA sequencing (RNAseq) data from CoMMpass dataset for responder/nonresponder prediction. The scores based on this seven‐gene expression in each sample were calculated and ranked. The survival data from 20% samples that ranked at each side of probability of response were compared; the samples on the “nonresponder” probability side have a shorter survival compared with the samples on the “responder” probability side. (D and E) The established model was also employed on mRNAseq data from the Mayo Clinic MM primary patient dataset. The scores were calculated in the samples that grouped by different stage and treatment protocols. (D) Analysis demonstrated that newly diagnosed (ND) patients’ samples more frequently have “responder” probabilities as compared to samples taken during therapy (other) or at refractory and end stages (ES). (E) Compared the patients with treatments (1 or 2 or >3 prior treatment protocols), the patients with no treatment or less treatment have more “responders” probabilities

Using the NanoString profiling data obtained from all ND (*n* = 52) and RR patients (*n* = 57), we further evaluated the predictive value of each differentially expressed gene for correlation with drug resistance and disease progression. Using edgeR software, we identified 45/121 differentially expressed genes between ND and RR samples (*p* ≤ 0.01) (Supporting Information Data 11). The correlation between those differentially expressed genes was identified by clustering analysis, for example, the expression of *PRR11* was found to cluster together with the expression of *BIRC5, CEP55*, *PBK*, and *DEPDC1* (Figure 5A). We identified 31/45 genes as significant predictors for separating ND from RR patients (*p* < 0.05, Figure 5B), with *CRBN*, *PRR11*, *CD53*, *BIRC5*, *DIRAS1*, *DEPDC1*, and *CEP55* being the most differentially expressed. Finally, using R‐package BhGLM, we built a multivariable ordinal model (MM‐IP‐7) that contained seven‐associated predictors, *CRBN*, C*EP55*, *DIRAS1*, SKA2, *CD53*, *PSMA7*, and *PSMD14* (Figure 6A). The performance of MM‐IP‐7 was evaluated by five‐fold cross‐validation resulting in an area under curve (AUC) = 0.91 (Figure 6B). Using MM‐IP‐7, we also analyzed RNAseq data from MMRF CoMMpass (*n* = 578) and Mayo Clinic MM patient registry (*n* = 487), found that model prediction correlated with OS (CoMMpass data, Figure 6C), disease stage, and treatment (Mayo Clinic MM patient data, Figure 6D,E). As expected, patient samples that classified as “responders” through the MM‐IP‐7 were enriched for longer OS, ND samples, samples without treatment, and fewer prior therapies.

## 4 DISCUSSION

We investigated the expression of 121 selected candidate genes that were previously demonstrated to associate with IMID and/or PI response to determine whether such genes could be combined into a biomarker assay of disease progression following combination chemotherapy regimens. We identified a subset of genes with changes in transcriptional expression during treatment and disease progression. The top downregulated gene in refractory and late stage disease was *CRBN*. We not only identified downregulation of *CRBN* in samples harvested during or after treatment with an IMiD‐based therapy, but also observed that downregulation became increasingly prominent in the relapsed and end stages samples. These results are consistent with both our own and other's prior reports, which demonstrated that with progressive IMiDs exposure, CRBN copy number and expression was reduced in MM cell lines and patient samples [7, 43]. Although several CRBN probes were designed to detect various isoforms of CRBN including the one with exon 10 deletion (associated with IMID resistance), no increased expression of this CRBN isoform was identified in resistant samples.

In addition to *CRBN*, in RR samples we also detected non‐*CRBN*‐related transcriptional changes identified in patient samples classified as nonresponders to pomalidomide (Supplemental Data 2) and in the XG1LenRes IMiD‐resistant isogenic HMCL, including downregulation of *IFITM1* and upregulation of *PRR11, BIRC5, PBK, DEPDC1*, *RNFT2*, and *CEP55. IFITM1* is one of the interferon‐stimulated genes upregulated by IMiD treatment and suggested to be involved in IMID‐mediated antimyeloma activity [44, 45]. Upregulation of *BIRC5*, *PRR11, PBK, DEPDC1*, *RNFT2*, and *CEP55* appeared subsequent to drug exposure in our study*. BIRC5* and *CEP55* are genes incorporated within the gene expression‐based proliferation indices [46], which is associated with poor prognosis. In the MMRF CoMMpass dataset, we found that high expression of *PRR11*, *PBK*, and *DEPDC1*, like *BIRC5*, correlated with short survival and poor drug response (Figure S5), implying upregulation of this group of genes may associate with disease progression and reduced drug response. Indeed, we demonstrated that inhibition of PBK reduced myeloma cell growth and enhanced lenalidomide and bortezomib activity in the IMiD‐resistant cell lines JJN3 and XG1LenRes (Figure S6). This result agrees with a recent study, demonstrating that inhibition of PBK overcame lenalidomide resistance [47]. Other upregulated genes identified in RR samples include *ETV4*. Upregulation of *ETV4* has been demonstrated in MM patient samples at the time of acquire IMiD resistance [14].

Based on the NanoString profiling data from ND and RR samples, we identified a subset of genes (Figure 5B) whose transcriptional expression may serve as predictive biomarkers for treatment nonresponse and disease progression, including several known genes involved in IMiD and PI sensitivity, including *CRBN* [11], *CD53* [32], and *PSMD4* [48]. We also built a multivariate ordinal model, MM‐IP‐7, for predicting drug resistance and disease progression. We demonstrated that classification into responder or nonresponder from this model correlated well with treatment and disease stage from a clinical dataset encompassing over 400 patients, suggesting that this model may have potential to monitor the emergence of drug resistance and disease progression. Interestingly, we also found that the MM‐IP‐7 prediction, in ND patients, is associated with OS, suggesting it may be used to identify patients that will be less responsive with faster progression. We recognize that a potential limitation of our study is that end stage patients were treated with combinations of multiple drugs, rather than a uniformly treated cohort; therefore some of our observed gene expression changes may be also linked to multiple‐drug resistance during disease progression. It will be important to extend our study to test more samples harvested at early relapse, especially from uniform therapy.

In the current study, we also demonstrated that NanoString nCounter technology is a reliable method for accurately detecting gene expression in MM samples. As described in non‐Hodgkin's lymphoma [23–25, 49], this method requires a small amount of RNA, is reasonably fast, and adaptable to a clinical diagnostic laboratory suggesting its potential application in MM bio‐marker development.

In summary, we developed a NanoString gene expression profiling‐based prediction model, which may serve as a useful tool for clinical investigation and therapy selection. This is the first study to build a multivariate ordinal model to predict clinical drug resistance, poor survival, and disease progression following treatment with IMIDs and PI, by using a comprehensive gene list generated from multiple other publications and the robust NanoString technology, which is currently in clinical practice.

## CONFLICT OF INTEREST

AKS has a consulting role for Amgen, Bristol‐Myers Squibb, Glaxo Smith Klein, Janssen, Ono, Regeneron, Skyline Diagnostics, and Tempus Inc. and has received research funding from Amgen, Celgene, and Janssen. The remaining authors declare no competing financial interests.

## Supporting information

Supporting information

Supporting information

Supporting information

## References

[1] Ito T , Ando H , Suzuki T , Ogura T , Hotta K , Imamura Y , et al. Identification of a primary target of thalidomide teratogenicity. Science. 2010;327(5971):1345–50. 20223979 10.1126/science.1177319

[2] Zhu YX , Braggio E , Shi CX , Bruins LA , Schmidt JE , Van Wier S , et al. Cereblon expression is required for the antimyeloma activity of lenalidomide and pomalidomide. Blood. 2011;118(18):4771–9. 21860026 10.1182/blood-2011-05-356063 PMC3208291

[3] Lopez‐Girona A , Mendy D , Ito T , Miller K , Gandhi AK , Kang J , et al. Cereblon is a direct protein target for immunomodulatory and antiproliferative activities of lenalidomide and pomalidomide. Leukemia. 2012;26(11):2326–35. 22552008 10.1038/leu.2012.119 PMC3496085

[4] Kronke J , Udeshi ND , Narla A , Grauman P , Hurst SN , McConkey M , et al. Lenalidomide causes selective degradation of IKZF1 and IKZF3 in multiple myeloma cells. Science. 2014;343(6168):301–5. 24292625 10.1126/science.1244851 PMC4077049

[5] Lu G , Middleton RE , Sun H , Naniong M , Ott CJ , Mitsiades CS , et al. The myeloma drug lenalidomide promotes the cereblon‐dependent destruction of Ikaros proteins. Science. 2014;343(6168):305–9. 24292623 10.1126/science.1244917 PMC4070318

[6] Kortum KM , Mai EK , Hanafiah NH , Shi CX , Zhu YX , Bruins L , et al. Targeted sequencing of refractory myeloma reveals a high incidence of mutations in CRBN and Ras pathway genes. Blood. 2016;128(9):1226–33. 27458004 10.1182/blood-2016-02-698092 PMC5524534

[7] Zhu YX , Shi CX , Bruins LA , Wang X , Riggs DL , Porter B , et al. Identification of lenalidomide resistance pathways in myeloma and targeted resensitization using cereblon replacement, inhibition of STAT3 or targeting of IRF4. Blood Cancer J. 2019;9(2):19. 30741931 10.1038/s41408-019-0173-0 PMC6370766

[8] Ocio EM , Fernandez‐Lazaro D , San‐Segundo L , Lopez‐Corral L , Corchete LA , Gutierrez NC , et al. In vivo murine model of acquired resistance in myeloma reveals differential mechanisms for lenalidomide and pomalidomide in combination with dexamethasone. Leukemia. 2015;29(3):705–14. 25102946 10.1038/leu.2014.238

[9] Dimopoulos K , Sogaard Helbo A , Fibiger Munch‐Petersen H , Sjo L , Christensen J , Sommer Kristensen L , et al. Dual inhibition of DNMTs and EZH2 can overcome both intrinsic and acquired resistance of myeloma cells to IMiDs in a cereblon‐independent manner. Mol Oncol. 2018;12(2):180–95. 29130642 10.1002/1878-0261.12157 PMC5792743

[10] Franssen LE , Nijhof IS , Couto S , Levin MD , Bos GMJ , Broijl A , et al. Cereblon loss and up‐regulation of c‐Myc are associated with lenalidomide resistance in multiple myeloma patients. Haematologica. 2018;103(8):e368–71. 29545338 10.3324/haematol.2017.186601 PMC6068039

[11] Schuster SR , Kortuem KM , Zhu YX , Braggio E , Shi CX , Bruins LA , et al. The clinical significance of cereblon expression in multiple myeloma. Leuk Res. 2014;38(1):23–8. 24129344 10.1016/j.leukres.2013.08.015 PMC3905958

[12] Zhou N , Gutierrez‐Uzquiza A , Zheng XY , Chang R , Vogl DT , Garfall AL , et al. RUNX proteins desensitize multiple myeloma to lenalidomide via protecting IKZFs from degradation. Leukemia. 2019;33(8):2006–21. 30760870 10.1038/s41375-019-0403-2 PMC6687534

[13] Sebastian S , Zhu YX , Braggio E , Shi CX , Panchabhai SC , Van Wier SA , et al. Multiple myeloma cells' capacity to decompose H2O2 determines lenalidomide sensitivity. Blood. 2017;129(8):991–1007. 28028022 10.1182/blood-2016-09-738872 PMC5324717

[14] Neri P , Tagoug I , Maity R , Stein CK , Kong M , Keats J , et al. Transcriptional plasticity compensates for Ikaros and Aiolos proteasomal degradation and mediates resistance to IMiDs in multiple myeloma (MM). Blood. 2017;130:63.

[15] Kale AJ , Moore BS . Molecular mechanisms of acquired proteasome inhibitor resistance. J Med Chem. 2012;55(23):10317–27. 22978849 10.1021/jm300434z PMC3521846

[16] Ling SC , Lau EK , Al‐Shabeeb A , Nikolic A , Catalano A , Iland H , et al. Response of myeloma to the proteasome inhibitor bortezomib is correlated with the unfolded protein response regulator XBP‐1. Haematologica. 2012;97(1):64–72. 21993678 10.3324/haematol.2011.043331 PMC3248932

[17] Nikesitch N , Ling SC . Molecular mechanisms in multiple myeloma drug resistance. J Clin Pathol. 2016;69(2):97–101. 26598624 10.1136/jclinpath-2015-203414 PMC4752637

[18] Shi CX , Kortum KM , Zhu YX , Bruins LA , Jedlowski P , Votruba PG , et al. CRISPR genome‐wide screening identifies dependence on the proteasome subunit PSMC6 for bortezomib sensitivity in multiple myeloma. Mol Cancer Ther. 2017;16(12):2862–70. 28958990 10.1158/1535-7163.MCT-17-0130 PMC5796678

[19] Barrio S , Stuhmer T , Da‐Via M , Barrio‐Garcia C , Lehners N , Besse A , et al. Spectrum and functional validation of PSMB5 mutations in multiple myeloma. Leukemia. 2019;33(2):447–56. 30026573 10.1038/s41375-018-0216-8

[20] Soriano GP , Besse L , Li N , Kraus M , Besse A , Meeuwenoord N , et al. Proteasome inhibitor‐adapted myeloma cells are largely independent from proteasome activity and show complex proteomic changes, in particular in redox and energy metabolism. Leukemia. 2016;30(11):2198–207. 27118406 10.1038/leu.2016.102 PMC5097071

[21] Besse A , Stolze SC , Rasche L , Weinhold N , Morgan GJ , Kraus M , et al. Carfilzomib resistance due to ABCB1/MDR1 overexpression is overcome by nelfinavir and lopinavir in multiple myeloma. Leukemia. 2018;32(2):391–401. 28676669 10.1038/leu.2017.212 PMC5808083

[22] Kulkarni MM . Digital multiplexed gene expression analysis using the NanoString nCounter system. Curr Protoc Mol Biol. 2011. 10.1002/0471142727.mb25b10s94. 21472696

[23] Scott DW , Wright GW , Williams PM , Lih CJ , Walsh W , Jaffe ES , et al. Determining cell‐of‐origin subtypes of diffuse large B‐cell lymphoma using gene expression in formalin‐fixed paraffin‐embedded tissue. Blood. 2014;123(8):1214–7. 24398326 10.1182/blood-2013-11-536433 PMC3931191

[24] Veldman‐Jones MH , Lai Z , Wappett M , Harbron CG , Barrett JC , Harrington EA , et al. Reproducible, quantitative, and flexible molecular subtyping of clinical DLBCL samples using the NanoString nCounter system. Clin Cancer Res. 2015;21(10):2367–78. 25301847 10.1158/1078-0432.CCR-14-0357

[25] Robetorye RS , Ramsower CA , Rosenthal AC , Yip TK , Wendel Spiczka AJ , Glinsmann‐Gibson BJ , et al. Incorporation of digital gene expression profiling for cell‐of‐origin determination (Lymph2Cx Testing) into the routine work‐up of diffuse large B‐cell lymphoma. J Hematop. 2019;12(1):3–10. 34447482 10.1007/s12308-019-00344-0 PMC8386504

[26] Eichner R , Heider M , Fernandez‐Saiz V , van Bebber F , Garz AK , Lemeer S , et al. Immunomodulatory drugs disrupt the cereblon‐CD147‐MCT1 axis to exert antitumor activity and teratogenicity. Nat Med. 2016;22(7):735–43. 27294876 10.1038/nm.4128

[27] Riggs DL , Herzog C , Garbitt VM , Keane N , Hillukka CJ , Hammond ZJ , et al. MiDs and BET inhibitors target distinct pathways of MYC dysregulation by super‐enhancers in multiple myeloma. Cancer Res. 2019;79:3015.

[28] Zhu YX , Braggio E , Shi CX , Kortuem KM , Bruins LA , Schmidt JE , et al. Identification of cereblon‐binding proteins and relationship with response and survival after IMiDs in multiple myeloma. Blood. 2014;124(4):536–45. 24914135 10.1182/blood-2014-02-557819 PMC4110660

[29] An J , Ponthier CM , Sack R , Seebacher J , Stadler MB , Donovan KA , et al. pSILAC mass spectrometry reveals ZFP91 as IMiD‐dependent substrate of the CRL4(CRBN) ubiquitin ligase. Nat Commun. 2017;8:15398. 28530236 10.1038/ncomms15398 PMC5458144

[30] Chapman MA , Sive J , Ambrose J , Roddie C , Counsell N , Lach A , et al. RNA‐seq of newly diagnosed patients in the PADIMAC study leads to a bortezomib/lenalidomide decision signature. Blood. 2018;132(20):2154–65. 30181174 10.1182/blood-2018-05-849893 PMC6310235

[31] Pelham RJ , Hu X , Moreau P , Oriol A , Quach H , Kovacsovics T , et al. Genomic predictors of progression‐free survival among patients with relapsed or refractory multiple myeloma treated with carfilzomib and dexamethasone or bortezomib and dexamethasone in the phase 3 endeavor trial. Blood. 2017;130:839.28818975

[32] Mitra AK , Mukherjee UK , Harding T , Jang JS , Stessman H , Li Y , et al. Single‐cell analysis of targeted transcriptome predicts drug sensitivity of single cells within human myeloma tumors. Leukemia. 2016;30(5):1094–102. 26710886 10.1038/leu.2015.361

[33] Shi CX , Zhu YX , Bruins L , Campos CBD , Stewart W , Braggio E , et al. The contribution of proteasome subunits to myeloma cell viability and proteasome inhibitor sensitivity. Blood. 2019;134:4337.

[34] Keats JK , Chesi M , Kuehl WM , Bergsagel PL . A simple and reliable method to verify the authenticity and purity of human myeloma cell lines. Blood. 2007;110:2485.

[35] Maity R , Neri PE , Tagoug I , Ren L , Slaby J , Jimenez‐Zepeda VH , et al. Cereblon (CRBN) splice isoform lacking exon 10 attenuates lenalidomide‐mediated degradation of Aiolos and is upregulated in immunomodulatory drugs (IMiDs) resistant myeloma (MM) patients. Blood. 2014;124:639.

[36] Lacy MQ , Hayman SR , Gertz MA , Dispenzieri A , Buadi F , Kumar S , et al. Pomalidomide (CC4047) plus low‐dose dexamethasone as therapy for relapsed multiple myeloma. J Clin Oncol. 2009;27(30):5008–14. 19720894 10.1200/JCO.2009.23.6802

[37] Lacy MQ , Hayman SR , Gertz MA , Short KD , Dispenzieri A , Kumar S , et al. Pomalidomide (CC4047) plus low dose dexamethasone (Pom/dex) is active and well tolerated in lenalidomide refractory multiple myeloma (MM). Leukemia. 2010;24(11):1934–9. 20827286 10.1038/leu.2010.190 PMC2978257

[38] Robinson MD , McCarthy DJ , Smyth GK . edgeR: a Bioconductor package for differential expression analysis of digital gene expression data. Bioinformatics. 2010;26(1):139–40. 19910308 10.1093/bioinformatics/btp616 PMC2796818

[39] Suzuki R , Shimodaira H . Pvclust: an R package for assessing the uncertainty in hierarchical clustering. Bioinformatics. 2006;22(12):1540–2. 16595560 10.1093/bioinformatics/btl117

[40] Yi N , Tang Z , Zhang X , Guo B . BhGLM: Bayesian hierarchical GLMs and survival models, with applications to genomics and epidemiology. Bioinformatics. 2019;35(8):1419–21. 30219850 10.1093/bioinformatics/bty803 PMC7963076

[41] Zhang Z . Variable selection with stepwise and best subset approaches. Ann Transl Med. 2016;4(7):136. 27162786 10.21037/atm.2016.03.35 PMC4842399

[42] Bonolo de Campos CMN , Petit JL , Polito AN , Zhu YX , Wang P , Bruins LA , et al. “Direct to drug” screening as a precision medicine tool in multiple myeloma. Blood Cancer J. 2020;10:54. 32393731 10.1038/s41408-020-0320-7 PMC7214452

[43] Gooding S , Ansari‐Pour N , Towfic F , Ortiz Estevez M , Chamberlain PP , Tsai KT , et al. Multiple cereblon genetic changes are associated with acquired resistance to lenalidomide or pomalidomide in multiple myeloma. Blood. 2021;137(2):232–7. 33443552 10.1182/blood.2020007081 PMC7893409

[44] Havens CG , Bjorklund C , Kang J , Ortiz M , Fontanillo C , Amatangelo M , et al. IMiDs immunomodulatory agents regulate interferon‐stimulated genes through cereblon‐mediated Aiolos destruction in multiple myeloma (MM) cells: identification of a novel mechanism of action and pathway for resistance. Blood. 2014;124(21):3432.

[45] Fedele PL , Willis SN , Liao Y , Low MS , Rautela J , Segal DH , et al. IMiDs prime myeloma cells for daratumumab‐mediated cytotoxicity through loss of Ikaros and Aiolos. Blood. 2018;132(20):2166–78. 30228232 10.1182/blood-2018-05-850727

[46] Hose D , Reme T , Hielscher T , Moreaux J , Messner T , Seckinger A , et al. Proliferation is a central independent prognostic factor and target for personalized and risk‐adapted treatment in multiple myeloma. Haematologica. 2011;96(1):87–95. 20884712 10.3324/haematol.2010.030296 PMC3012769

[47] de Boussac H , Bruyer A , Jourdan M , Maes A , Robert N , Gourzones C , et al. Kinome expression profiling to target new therapeutic avenues in multiple myeloma. Haematologica. 2020;105(3):784–95. 31289205 10.3324/haematol.2018.208306 PMC7049359

[48] Shaughnessy JD Jr. , Qu P , Usmani S , Heuck CJ , Zhang Q , Zhou Y , et al. Pharmacogenomics of bortezomib test‐dosing identifies hyperexpression of proteasome genes, especially PSMD4, as novel high‐risk feature in myeloma treated with Total Therapy 3. Blood. 2011;118(13):3512–24. 21628408 10.1182/blood-2010-12-328252 PMC3186329

[49] ` Mottok A , Wright G , Rosenwald A , Ott G , Ramsower C , Campo E , et al. Molecular classification of primary mediastinal large B‐cell lymphoma using routinely available tissue specimens. Blood. 2018;132(22):2401–5. 30257882 10.1182/blood-2018-05-851154 PMC6265647

