# Supplementary material for: Transcriptional profiles define drug refractory disease in myeloma
Source: EJHaem. 2022 May 9;3(3):804–14. doi: 10.1002/jha2.455 (PMC9422020; doi:10.1002/jha2.455)
Supplement: Supplementary file 1 — Supporting information [file JHA2-3-804-s001.pdf]

Resistant cell lines vs. baseline of sensitive cell lines (4 pairs)

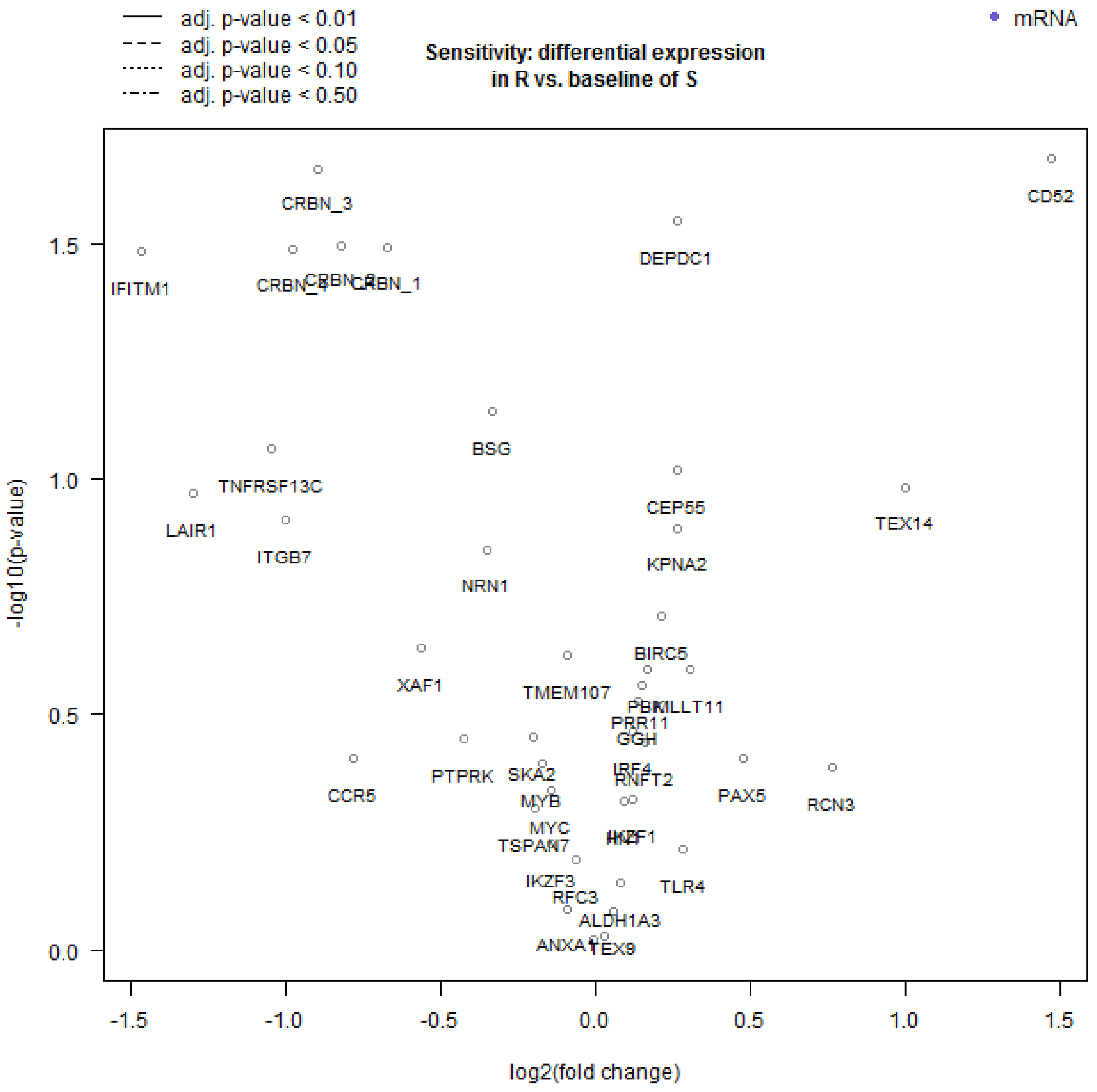

mRNA	Log2 fold change	P-value
CD52	1.47	0.0209
CRBN_3	-0.899	0.0219
DEPDC1	0.264	0.0283
CRBN_2	-0.826	0.0318
CRBN_1	-0.673	0.0322
CRBN_4	-0.979	0.0326
IFITM1	-1.47	0.0329
BSG	-0.333	0.0717
TNFRSF13C	-1.05	0.0863
CEP55	0.263	0.0956
TEX14	1	0.104
LAIR1	-1.3	0.107
ITGB7	-1	0.122
KPNA2	0.263	0.127
NRN1	-0.351	0.141

KMS11
KMS11LenRes

MM1.S
MM1.S Len Res

OPM2
OPM2Len Res

XG1
XG1LenRes

Supplemental figure 1

Paired PIs vs ND samples (3 pairs)

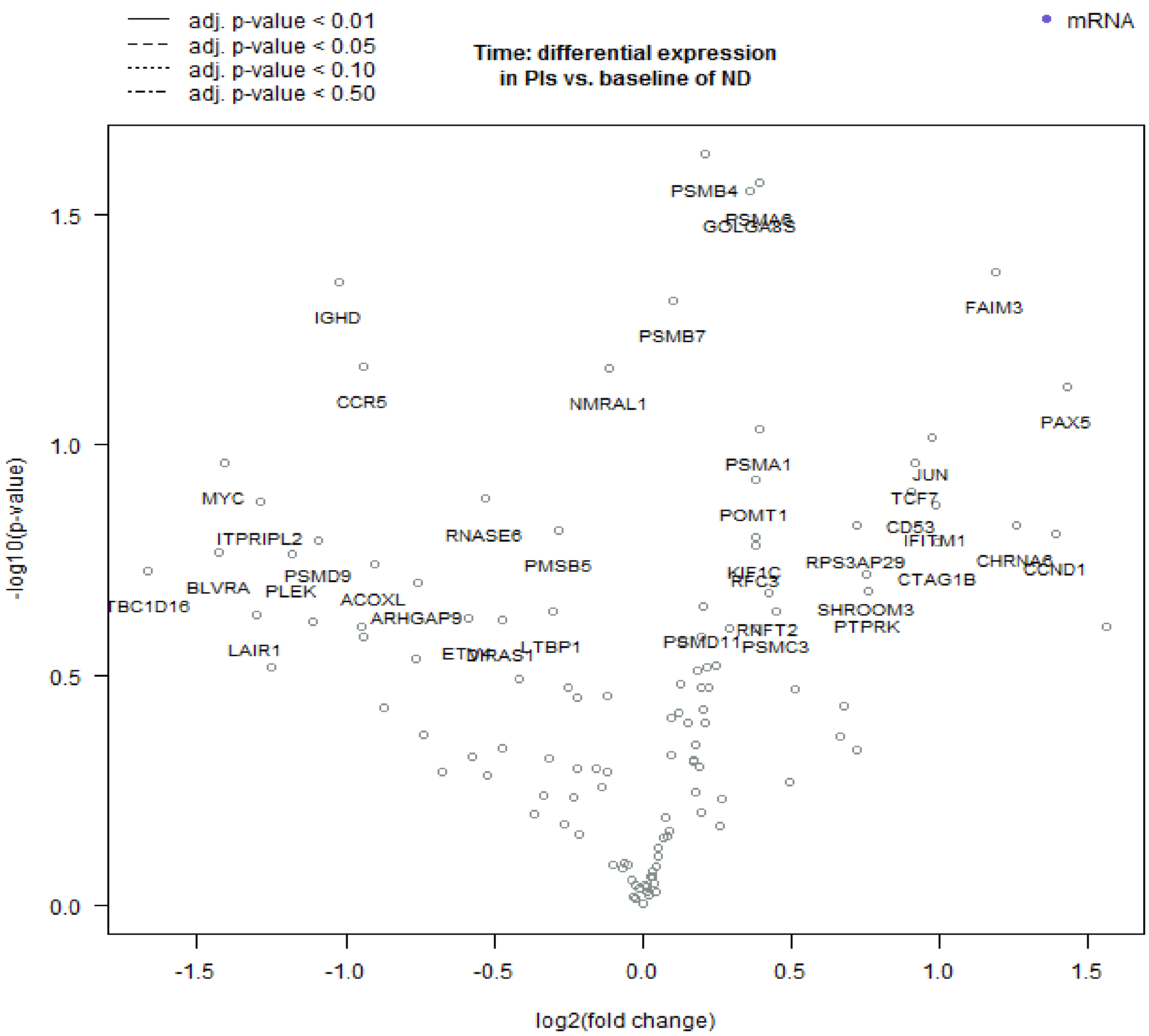

mRNA	Log2 fold change	P-value
PSMB4	0.21	0.0233
PSMA6	0.395	0.0269
GOLGA8S	0.361	0.028
FAIM3	1.19	0.042
IGHD	-1.02	0.0441
PSMB7	0.102	0.0484
CCR5	-0.939	0.0674
NMRAL1	-0.115	0.0678
PAX5	1.43	0.0745
PSMA1	0.393	0.0922
JUN	0.972	0.0965
TCF7	0.917	0.109
MYC	-1.41	0.109
POMT1	0.378	0.119
CD53	0.907	0.126

Supplemental figure 2

Paired late samples vs early samples (5 pairs)

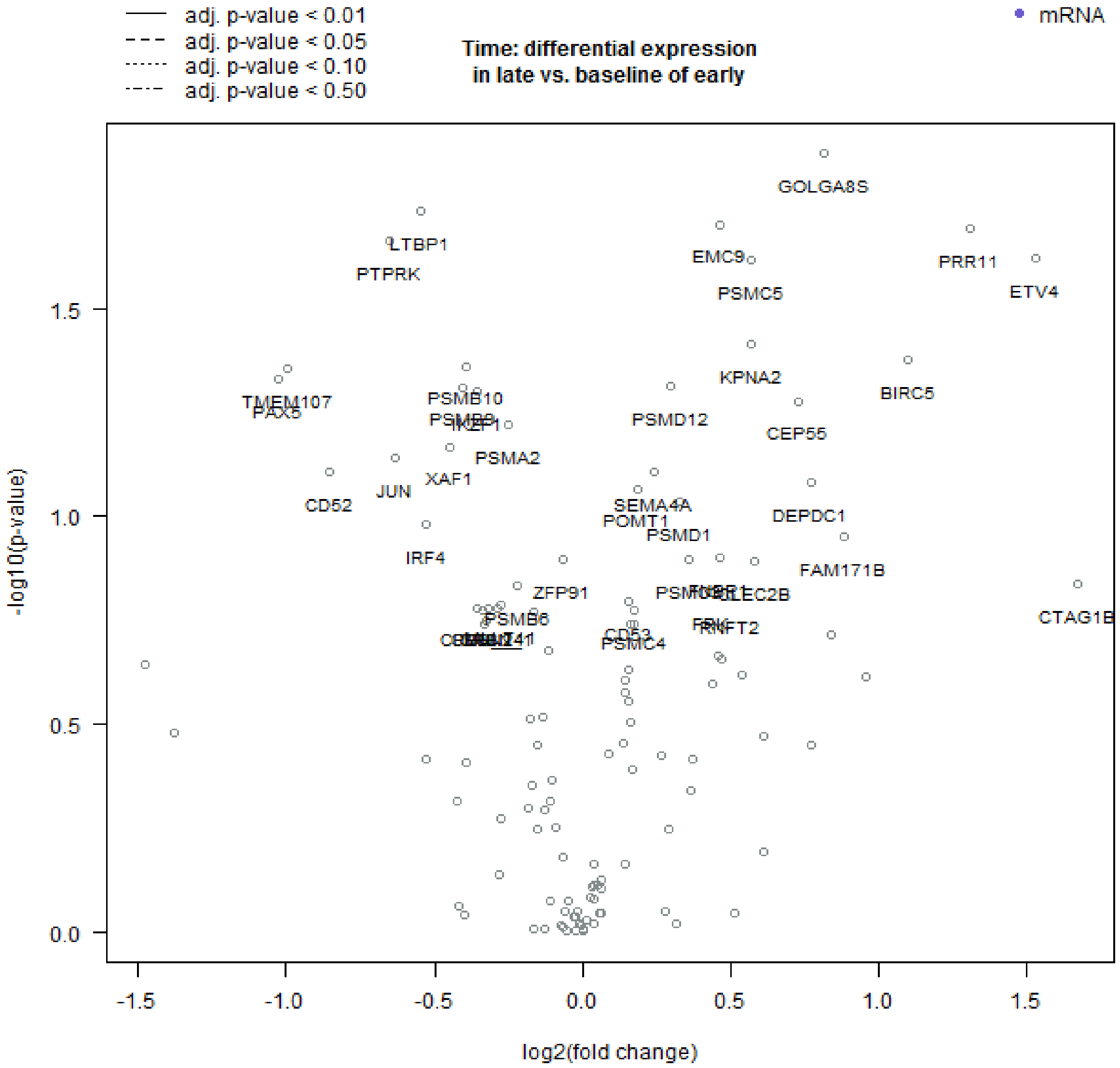

mRNA	Log2 fold change	P-value
GOLGA8S	0.818	0.0134
LTBP1	-0.546	0.0184
EMC9	0.463	0.0198
PRR11	1.31	0.0203
PTPRK	-0.654	0.0218
ETV4	1.53	0.024
PSMC5	0.568	0.0241
KPNA2	0.571	0.0385
BIRC5	1.1	0.0421
PSMB10	-0.395	0.0435
TMEM107	-0.997	0.0441
PAX5	-1.03	0.0469
PSMD12	0.298	0.0487
PSMB9	-0.406	0.0489
IKZF1	-0.357	0.05

Supplemental figure 3

A

Sensitive HMCLs:
KMS11
MM1.S
H929
XG1
OPM2
KMS34

Resistant HMCLs:
FR4
JJN3
RPMI/8226
OCIMY5
SKMM2
EJM
KMS12PE
KMS26

HMCLs: IMiD resistant (n=8) vs sensitive cell lines (n=6)

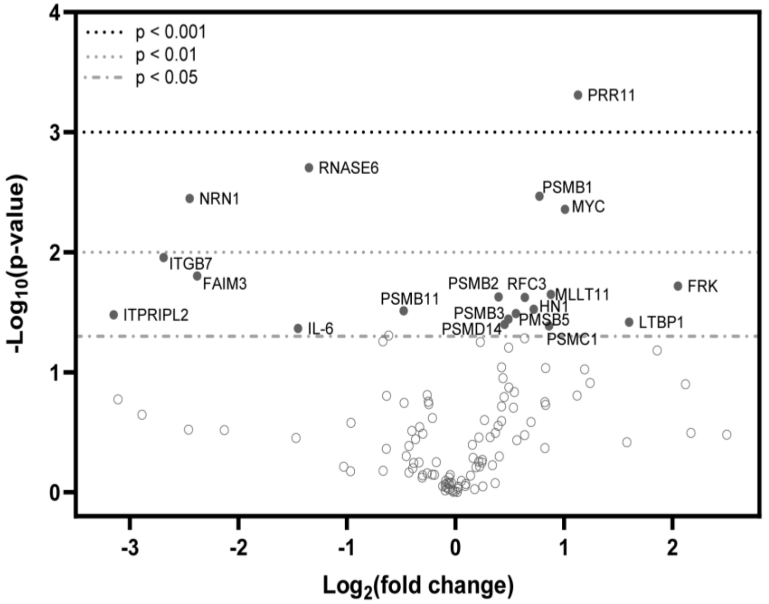

mRNAs	Log2 fold change	P-value
PRR11	1.13	0.000491
RNASE6	-1.35	0.00198
PSMB1	0.773	0.00341
NRN1	-2.45	0.00356
MYC	1.01	0.0044
ITGB7	-2.69	0.0111
FAIM3	-2.38	0.0158
FRK	2.05	0.0191
MLLT11	0.879	0.0224
PSMB2	0.397	0.0236
RFC3	0.64	0.0238
HN1	0.721	0.0298
PSMB11	-0.478	0.0307
PSMB5	0.558	0.0325
ITPRIPL2	-3.15	0.0332
PSMB3	0.488	0.0361

B

MM1.S
MM1.S BorRes
IRF4
IRF4 BorRes
JJN3
JJN3 BorRes
U266
U266 BorRes
RPMI/8226
RPMI/8226 BorRes

HMCLs: Bortezomib resistant vs sensitive cell lines (n=5 pairs)

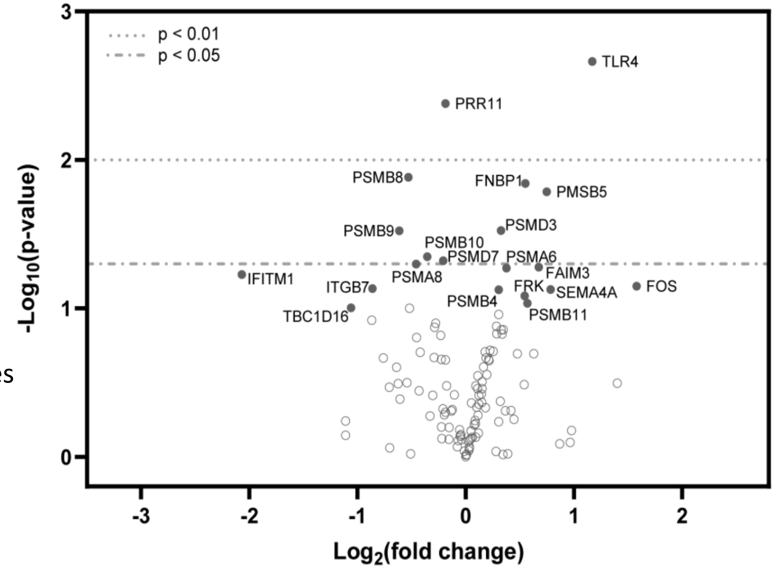

mRNAs	Log2 fold change	P-value
TLR4	1.17	0.00217
PRR11	-0.186	0.00417
PSMB8	-0.53	0.0131
FNBP1	0.552	0.0144
PSMB5	0.748	0.0164
PSMD3	0.326	0.0299
PSMB9	-0.613	0.03
PSMB10	-0.356	0.0449
PSMD7	-0.209	0.0477
PSMA8	-0.458	0.0503
FAIM3	0.676	0.0527
PSMA6	0.376	0.0535
IFITM1	-2.07	0.0592
FOS	1.58	0.0708
ITGB7	-0.862	0.0737
SEMA4A	0.785	0.0746

Supplemental figure 4

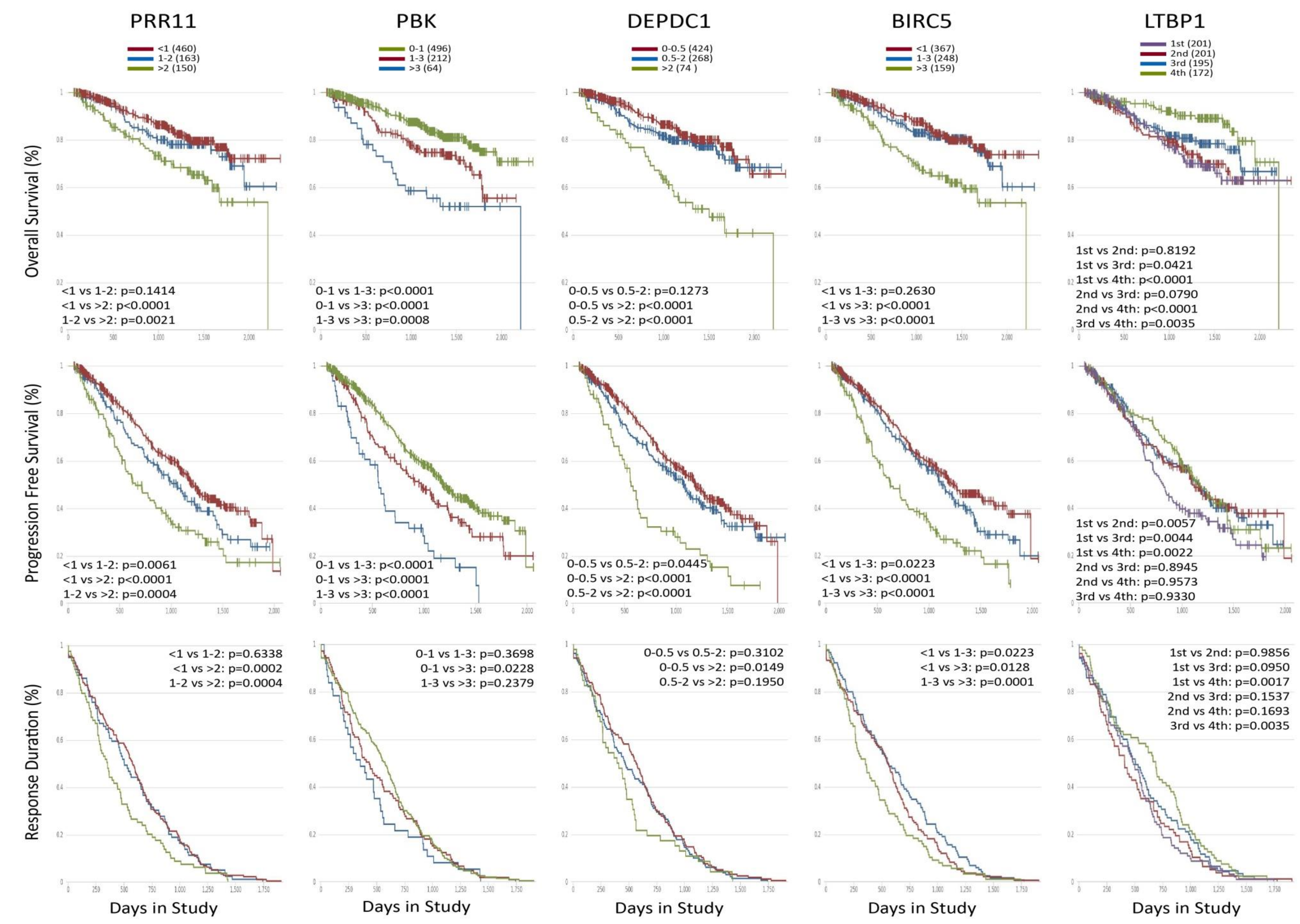

Supplementary figure 5

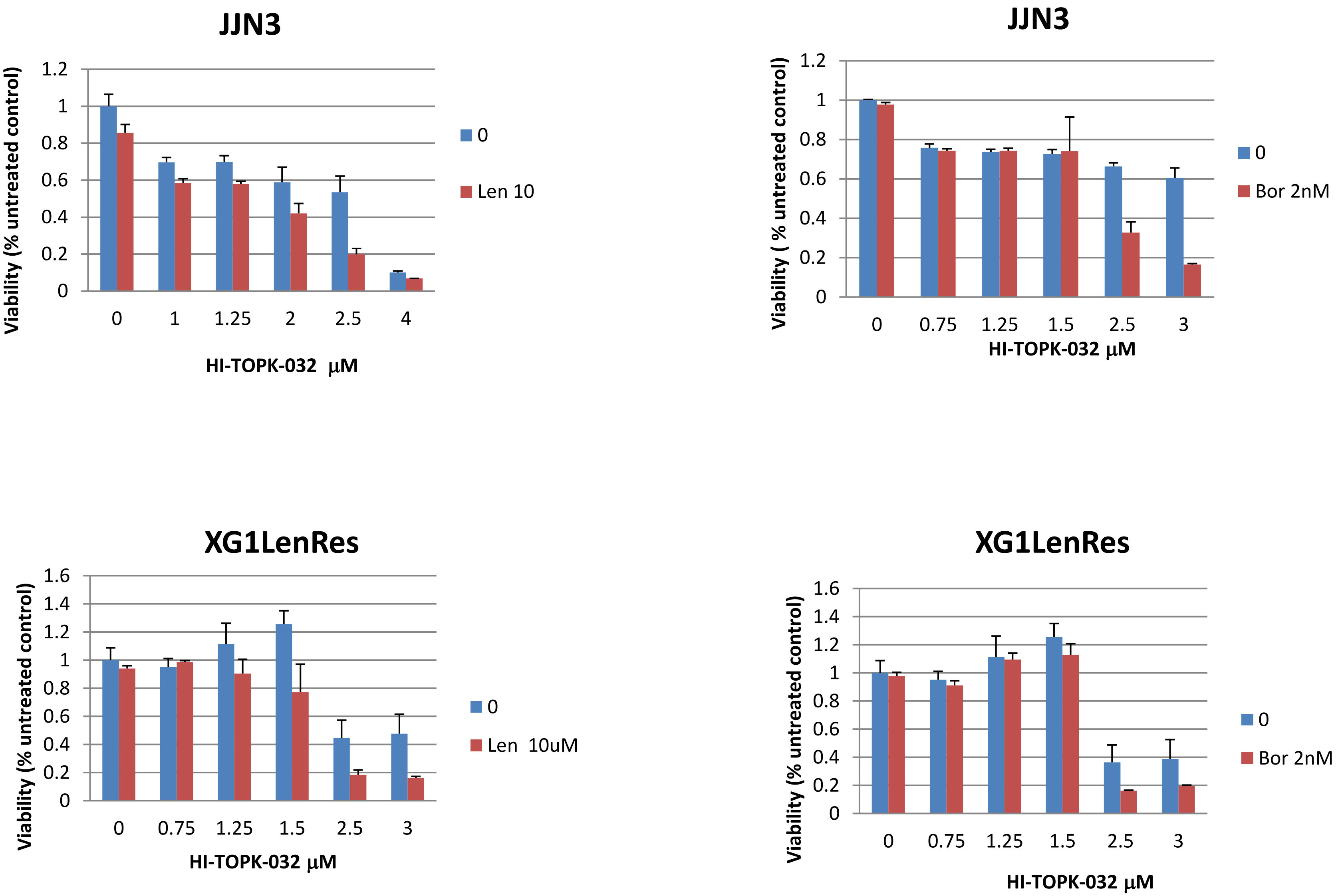

Supplemental figure 6
