# Supplementary material for: Transcriptional profiles define drug refractory disease in myeloma
Source: EJHaem. 2022 May 9;3(3):804–14. doi: 10.1002/jha2.455 (PMC9422020; doi:10.1002/jha2.455)
Supplement: Supplementary file 2 — Supporting information [file JHA2-3-804-s002.docx]

**Figure legends**

Supplementary figure 1. **Detection of differentially expressed genes between 4 pairs of IMIDs sensitive and resistant isogenic HMCLs**

Volcano plot displaying each gene's -log_10_ (p-value) and log_2_ fold change with the selected covariate. Highly statistically significant genes fall at the top of the plot above the horizontal lines, and highly differentially expressed genes fall to either side. Horizontal lines indicate various False Discovery Rate (FDR) thresholds or p-value thresholds if there is no adjustment to the p-values. The 40 most statistically significant genes are labeled in the plot. Top 15 differentially expressed genes are shown in the table.

Supplementary figure 2. **Detection of differentially expressed genes between newly diagnosed MM and samples harvested during active treatment with PIs**

Volcano plot displaying each gene's -log_10_ (p-value) and log_2_ fold change with the selected covariate. Highly statistically significant genes fall at the top of the plot above the horizontal lines, and highly differentially expressed genes fall to either side. Horizontal lines indicate various False Discovery Rate (FDR) thresholds or p-value thresholds if there is no adjustment to the p-values. The 40 most statistically significant genes are labeled in the plot. Top 15 differentially expressed genes are shown in the table. The comparison was made between 3 paired samples harvested at newly diagnosed and during or after treatment with PIs based therapy (no IMIDs were used).

Supplementary figure 3. **Detection of differentially expressed genes between the samples collected at early and late stage of disease**

Volcano plot displaying each gene's -log_10_ (p-value) and log_2_ fold change with the selected covariate. Highly statistically significant genes fall at the top of the plot above the horizontal lines, and highly differentially expressed genes fall to either side. Horizontal lines indicate various False Discovery Rate (FDR) thresholds or p-value thresholds if there is no adjustment to the p-values. The 40 most statistically significant genes are labeled in the plot. Top 15 differentially expressed genes are shown in the table. The samples collected at early time points were compared with the samples collected at later time points from five MM patients.

Supplementary figure 4. **Detection of differentially expressed genes between immune modulator or proteasome inhibitor sensitive and resistant HMCLs.**

Volcano plot displaying each gene's -log_10_ (p-value) and log_2_ fold change with the selected covariate. Highly statistically significant genes fall at the top of the plot above the horizontal lines, and highly differentially expressed genes fall to either side. Horizontal lines indicate various p-value thresholds. The 20 most statistically significant genes are labeled in the plot. The top 16 differentially expressed genes are shown in the table. (A) Comparison of six lenalidomide resistant HMCLs with eight lenalidomide sensitive HMCLs. (B) five paired isogenic bortezomib sensitive and resistant HMCLs were compared.

Supplementary figure 5. **Correlation of the expression of selected genes with survival and drug response in MMRF CoMMpass data set**

The plots were prepared based on the RNAseq and clinical data in MMRF CoMMpass database (Explore 1A13). The Kaplan-Meier curves for selected clinical endpoints with censoring show the estimated probability over time for all patients in the dataset. The plot shows the differential probabilities between the patients belonging to the selected groups. A test of equal hazards between groups is performed and the p-value for the log-rank is displayed along with the hazard ratio between each pairwise groups.

Supplementary figure 6. **Analysis of MM cell growth and drug response after inhibition of PBK in HMCLs**

JJN3 and XG1LenRes, IMiD resistant cell lines, were treated with a PBK specific inhibitor, HI-TOPK-032 alone (Calbiochem) or combined with either lenalidomide (Len) or bortezomib (Bor) to evaluate synergy by the MTT assay. Inhibition of PBK activity using Hi-TOPK-032, enhanced both lenalidomide and bortezomib sensitivity in JJN3 and XG1LenRes cells
